# Symptomatic and Asymptomatic *Campylobacter* Infections Associated with Reduced Growth in Peruvian Children

**DOI:** 10.1371/journal.pntd.0002036

**Published:** 2013-01-31

**Authors:** Gwenyth Lee, William Pan, Pablo Peñataro Yori, Maribel Paredes Olortegui, Drake Tilley, Michael Gregory, Richard Oberhelman, Rosa Burga, Cesar Banda Chavez, Margaret Kosek

**Affiliations:** 1 Department of International Health, Johns Hopkins Bloomberg School of Public Health, Baltimore, Maryland, United States of America; 2 Duke Global Health Institute, Duke University, Durham, North Carolina, United States of America; 3 Biomedical Research, Asociación Benéfica PRISMA, Iquitos, Peru; 4 U.S. Naval Medical Research Unit Six, Lima, Peru; 5 Department of Tropical Medicine, Tulane School of Public Health, New Orleans, Louisiana, United States of America; University of California San Diego School of Medicine, United States of America

## Abstract

**Background:**

Although diarrheal illnesses are recognized as both a cause and effect of undernutrition, evidence for the effect of specific enteropathogens on early childhood growth remains limited. We estimated the effects of undernutrition as a risk factor for campylobacteriosis, as well as associations between symptomatic and asymptomatic *Campylobacter* infections and growth.

**Methodology/Principal Findings:**

Using data from a prospective cohort of 442 children aged 0–72 months, the effect of nutritional status on the incidence of *Campylobacter* infection was estimated using uni- and multivariate Poisson models. Multivariate regression models were developed to evaluate the effect of *Campylobacter* infection on weight gain and linear growth. Overall, 8.3% of diarrheal episodes were associated with *Campylobacter* (crude incidence rate = 0.37 episodes/year) and 4.9% of quarterly asymptomatic samples were *Campylobacter* positive. In univariate models, the incidence of *Campylobacter* infection was marginally higher in stunted than non-stunted children (IRR 1.270, 95% CI (0.960, 1.681)(p = 0.095). When recent diarrheal burdens were included in the analysis, there was no difference in risk between stunted and unstunted children. Asymptomatic and symptomatic *Campylobacter* infections were associated with reduced weight gain over a three-month period (65.5 g (95% CI: −128.0, −3.0)(p = 0.040) and 43.9 g (95% CI:−87.6, −1.0)(p = 0.049) less weight gain, respectively). Symptomatic *Campylobacter* infections were only marginally associated with reduced linear growth over a nine month period (−0.059 cm per episode, 95% CI: −0.118, 0.001)(p = 0.054), however relatively severe episodes were associated with reduced linear growth (−0.169 cm/episode, 95% CI −0.310, −0.028)(p = 0.019).

**Conclusions/Significance:**

Our findings suggest that *Campylobacter* is not as benign as commonly assumed, and that there is evidence to support expanding the indications for antibiotic therapy in campylobacteriosis in children.

## Author Summary

*Campylobacter* is a common cause of diarrheal disease among children at risk for growth failure in the developing world. We evaluated risk factors for *Campylobacter* infection as well as the association between symptomatic and asymptomatic *Campylobacter* infections and child growth over three and nine-month periods. Undernourished (stunted) children were more likely to experience a *Campylobacter* infection, but adjusting for a recent history of diarrheal disease attenuated this relationship. Both symptomatic and asymptomatic infections were associated with poorer weight gain and symptomatic *Campylobacter* infections were marginally associated with poorer linear growth on an order similar to what has been reported for other bacterial pathogens and less than what has been reported for some protozoal and parasitic infections. In a subset of severe infections that made up about twenty percent of total illnesses, the associations were poorer growth were of greater magnitude. *Campylobacter* infections are frequently viewed as benign, but our study suggests that this is not always the case. Rapid diagnostics for *Campylobacter jejuni* and *coli* could attenuate acquired linear growth deficits in populations where campylobacteriosis is highly endemic by facilitating improved case management.

## Introduction

*Campylobacter* is a common cause of diarrhea among infants and children in the developing world [1]. Risk factors include poor sanitation and close contact with animals [2]. Among the known species and subspecies of *Campylobacter*, *C. jejuni* is the most frequently isolated, followed by *C. coli* [3]. *Campylobacter*-associated diarrhea is generally acute but self-limiting [3]. Unlike other common bacterial causes of diarrheal disease, it is frequently isolated from stools up to weeks following an episode, with reported mean excretion times following diarrhea as long as 14 days [4], [5].

Asymptomatic *Campylobacter* infections are common among older children, and are associated with prolonged excretion in a significant percentage of cases [4]. In the developing world, asymptomatic *Campylobacter* carriage is more common among malnourished children; an observation that has been confirmed individually for *C. jejuni* [6], [7], and other species [8]. This has led to the suggestion that in this context it is an opportunistic infection, perhaps related to undernutrition-induced immunosuppression [8]. However, the relationship between infection and undernutrition is complex and the effect of *Campylobacter* infection and carriage on childhood growth has not to our knowledge previously been quantified using a longitudinal study design.

Biologically, there is reason to believe that *Campylobacter* infection may have an enduring impact on childhood growth. In addition to rarer immune-mediated long term sequelae including reactive arthritis and Guillain-Barré syndrome, *Campylobacter* is a risk factor for post-infectious inflammatory bowel syndrome and, more controversially, inflammatory bowel disease [9]–[12]. This is consistent with the hypothesis that there is an adverse physiologic insult extending beyond the period of acute diarrhea, at least in a subset of cases. *Campylobacter* infection has been found to affect epithelial barrier integrity [13], suggesting that prolonged excretion may be associated with persistent mucosal injury.

Our objectives were to evaluate undernutrition as a risk factor for *Campylobacter* infection and to estimate the effects of symptomatic and asymptomatic *Campylobacter* infections on early childhood growth. Longitudinal analysis methods were used to disentangle the potentially bi-directional relationship between undernutrition and *Campylobacter* in a population with high rates of diarrheal disease and chronic undernutrition [14].

## Methods

Data were from an age-stratified, prospective, community-based study of 442 children 0–72 months of age in a semi-rural community in the Peruvian Amazon, between 2002 and 2006 [14]. The cohort and study design were described previously [14], [15]. Briefly, recruitment, socioeconomic and demographic information were based on community censuses conducted before and during the study period. Every third child in a list of age-eligible children living in the study community without another family member enrolled was invited to participate. Therefore, only one child per household was enrolled in the study at any given time. Enrollment was continuous through the study period. The overall objective was to compare the associations between common etiologies of diarrhea and early childhood growth [14], [15], with the sample size based on this objective.

Length (children 0–23 months) or height (children 24–72 months) were measured using a marked platform with a sliding footboard, and weight was measured using Salter scales (Salter Housewares Ltd, Tonbridge, England). This occurred monthly on the day of their birth. For analyses, the measures were converted to z-scores (height-for-age-Z (HAZ) and weight-for-height-Z (WHZ)) using the 2006 WHO standards [16].

Participating families were visited three times weekly by a trained health promoter to document the number and consistency of stools passed by the child, as well as other symptoms like fever, and reported medication use, including specific antibiotics. This generated a continuous history of diarrheal disease for each child.

Diarrhea was defined by three or more semi-liquid stools reported over a 24-hour period, with episodes separated by at least three symptom-free days. Stool samples were collected as soon as possible after the case definition was met, and not more than two days after the episode ended. One sample was sought for all episodes; however, children who were culture-negative for *Campylobacter* and *Shigella* but continued to have diarrhea provided a sample every fourth day until the episode ended. Asymptomatic stool samples were collected quarterly to detect background enteropathogen carriage.

Fresh stool samples were placed in glycerol buffered saline and Cary-Blair medium and transported in a cooler from the field site for same-day plating. Briefly, one gram of feces in one milliliter glycerol buffered saline, pH 7·2, was received from the field and agitated briefly. A 100 ul aliquot was then placed on a 0.45 um sterile nitrocellulose filter overlying a Columbia blood agar plate and allowed to diffuse passively for 30 minutes. The filter was removed with sterile forceps and the plate was incubated in a microaerophilic atmosphere (5% O2, 10% CO2, 85% N) at 42 C for 48 hours. Suspected colonies were evaluated by gram staining, oxidase and catalase testing. Colonies confirmed as *Campylobacter* by these methods were then evaluated by the hippurate hydrolysis tube test to distinguish *Campylobacter jejuni* from *Campylobacter coli* The specimen aliquot in Cary-Blair was plated on MaConkey, Hektoen, XLD, and TCBS agar plates as previously reported [14]. Additionally, for all samples, technician-observed gross blood and mucus were noted. Methylene blue was used to identify fecal leukocytes, and occult blood was identified by Hemoccult fecal occult blood test (SmithKline Diagnostics, Inc., Sunnyvale, California).

In cases where the child was still symptomatic when bacteriological culture confirmed a positive *Campylobacter* (or other enteropathogen) result, appropriate antibiotic therapy was administered (azithromycin or erythromycin). In the case of *Campylobacter*, a 72-hour delay between specimen acquisition and microbiologic reporting meant that the majority of cases resolved before a result was received.

Written informed consent to participate in the study was provided by all subjects. Parents consented on behalf of their children. The study protocol was approved by the Institutional Review Boards of Johns Hopkins Bloomberg School of Public Health (Baltimore, MD), US Navy Medical Research Center (Silver Spring, MD), Asociacion Benefica PRISMA (Lima, Peru), and the Regional Health Department of Loreto Peru.

### Definitions and Descriptive Analyses

*Campylobacter* infection was defined as asympyomatic if there was not a diarrheal episode associated with *Campylobacter* in the two weeks period preceding its culture identification. Symptomatic campylobacteriosis was discounted if another *Campylobacter-*episode had been detected less than 15 days prior (i.e. it was taken to be a persistent infection).

**Figure 1:**
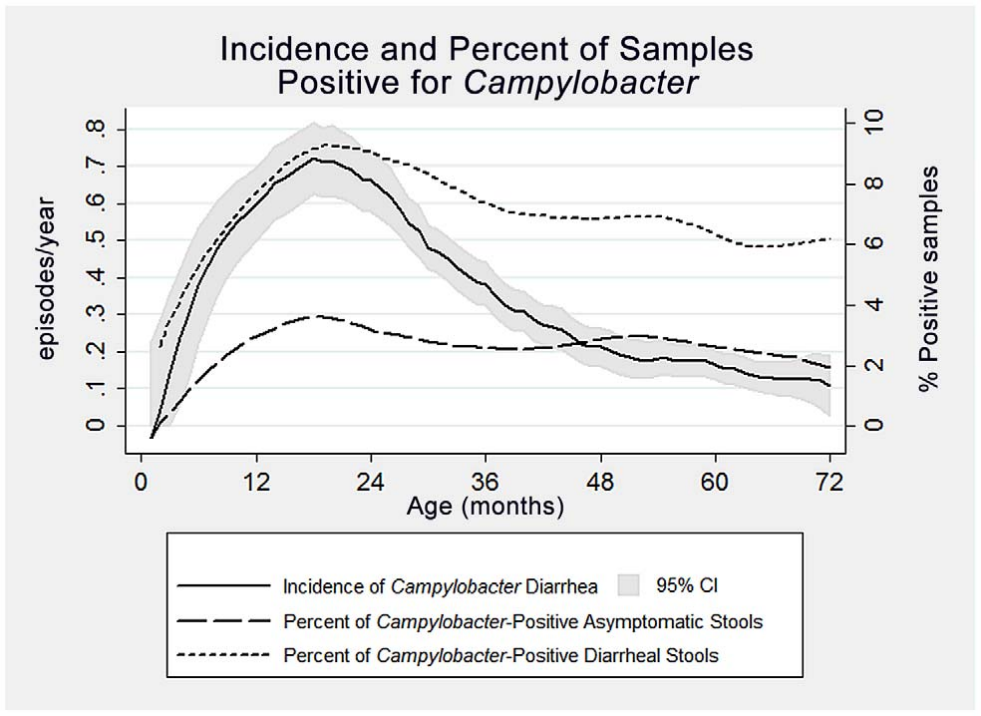
Smoothed plots of incidence versus age, and the percentage of stools positive by etiology. The peak incidence of *Campylobacter*-associated diarrhea occurs at approximately 18 months of age, and declines rapidly thereafter. However, its isolation rate in diarrheal and asymptomatic stool samples remains roughly constant from 18–72 months of age.

Baseline characteristics of children who experienced at least one case of *Campylobacter-*associated diarrhea during the course of the study were compared to children who did not experience any *Campylobacter-*associated diarrhea during the study period. Two-sided t-tests were used to compare the child's age of entry into the study and total time in the study, and multivariate poisson models adjusting for age were used to describe the incidence of non-campylobacter diarrhea. Running-average smoothed plots of incidence versus age, and the percentage of stools positive by etiology, were created (see Figure 1). Throughout the analyses, p-values of between 0.10 and 0.05 were considered to be “marginally significant”, p-values of less than 0.05 “significant”, and p-values of less than 0.01 “very significant”.

### Risk Factors for *Campylobacter* Infection

Bivariate Poisson models with a child-level random intercept to account for host-specific susceptibility were built to examine potential risk factors for campylobacteriosis and asymptomatic *Campylobacter*. Risk factors signficant at the p<0.15 level in univariate models were included in a multivariate model. Sex, age (using linear splines with knots at 18 and 36 months), non-*Campylobacter* diarrheal incidence in the past 3 months, campylobacteriosis in the past three months, seasonal effects (as sine/cosine terms), SES (as log per-capita income), maternal education, maternal age, the the presence of a household water connection, a private household latrine, poultry in the household, breast-feeding status, and birth weight, were considered for inclusion. The effects of prior undernutrition on the risk of campylobacteriosis was tested by considering several variables related to anthropometric status as measured in the month prior to the detection of *Campylobacter*. We considered HAZ (height-for-age-Z) and WHZ (weight-for-height-Z) as continuous independent variables, as well stunting as a binary variable (HAZ <−2), and WHZ categorized as less than −1, between −1 and 0, and greater than 0 (WHZ less than −2 was rare in this cohort). In final models, stunting as a binary variable and WHZ as a categorical variable were used. Correlations and kappa statistics comparing prior nutritional status and prior diarrheal disease were calculated.

### Effects of *Campylobacter* Infection on Growth

As it was assumed that deficits in weight gain would precede linear deficits, models were constructed to examine the effects of symptomatic and asymptomatic *Campylobacter* on weight over three month periods. Linear growth periods ranging from two to nine months were used to examine the effects of campylobacteriosis and asymptomatic *Campylobacter* infections on length/height. The longer interval was retained in the final models as we posited that durable linear growth deficits were of increased importance relative to short term deficits.

All models included a random intercept for each study child. The covariance structure was fixed such that the residuals took a third-order moving average form for change-in-weight models, and a first-order autoregressive form for change-in-height models. This choice was based on a visual inspection of the autocorrelation function as well as model fit. Seasonal effects were considered by including the terms 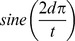 and 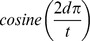, where *d* is the day of the year and *t* is 365 [17], [18]. Per-capita income [19], prior nutritional status (stunting and WHZ category as defined above), sex, birth weight, maternal age and education, and breast-feeding status, were also considered for inclusion. Variables were selected into the final model based on model fit.

Age terms were included as fractional polynomials, a method to adjust for curvi-linear relationships. [15], [20] These terms were set separately for each model.

The final models are shown in Equations 1 and 2. Two sets of models were constructed. The first estimates the association of symptomatic and asymptomatic *Campylobacter* on change in weight, after adjusting for stunting, WHZ category, birth weight, season, age and per-capita income (Equation 1). The second estimates the association of *Campylobacter* infection on length/height, controlling for the same factors (Equation 2).
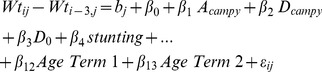
(1)In Equation 1, 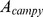 is a binary variable set to one if asymptomatic *Campylobacter* was encountered, and zero otherwise. Similarly, 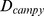 is one if campylobacteriosis is encountered (two cases of more than one symptomatic *Campylobacter* in a three-month period were re-classified to 1), and zero otherwise. 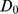 is a continuous variable representing the number of diarrheal episodes in the three months unassociated with *Campylobacter*. In weight models, anthropometry that was measured during or within two days of a *Campylobacter* associated diarrheal episode was excluded from the analysis, in order to reduce the possibility that reductions in weight were related to clinical or subclinical dehydration.
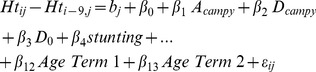
(2)In Equation 2, 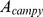 is a continuous variable taking values between one and three, the number of quarters in the nine month interval in which asymptomatic *Campylobacter* infection was observed. 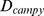 and 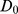 are continuous variables representing the total incidence of *Campylobacter*-associated and non- *Campylobacter*-associated episodes of diarrhea in the interval.

### Effects of Treated Versus Untreated Campylobacteriosis on Growth

A second set of models were created, identical to those described above, except that symptomatic *Campylobacter* infection was subdivided into two types: treated, and untreated, which were tested separately. Therefore, the incidence of treated and untreated infections were taken as two separate binary variables (in weight models), or two separate continous variables (in height models).

All analysis was performed using Stata 11 (Statacorp, College Station, TX).

## Results

Ninety-six percent of families invited to participate in the study consented (five families refused). Of those enrolled, 20.8% (N = 92) dropped out during the study (median time in study = 15 month); permanent migration outside the community was the principal reason. 442 children were enrolled, nine (2.0%) were excluded because of limited anthropometric data, defined as fewer than three consecutive months of anthropometry. In total, we conducted 838 total child-years of surveillance and collected anthropometric data during 10,985 study visits. Child characteristics are outlined in Table 1.

**Table 1:** Cohort characteristics.

		Mean ± SD or Percent
**Age at Enrollment, months [N = 433]**		27.3±20.9
**Follow-up time, months [N = 433]**		27.1±13.6
**Percent Sex, female [N = 433]**		51.5%
**Birth weight, kg [N = 405]**		3.1±0.5
**HAZ, at enrollment [N = 433]**		−1.7±0.7
**WHZ at enrollment [N = 433]**		0.7±1.1
**Mean Maternal age** * **, yrs [N = 385]**		23.7±7.4
**Mean Maternal height, cm [n = 394]**		149.0±5.6
**Maternal education [385]**	Primary or less	52.5%
	Some Secondary or more	47.5%
**Presence of Household Poultry [N = 396]**		29.80%
**Family has private latrine [N = 409]**		65.5%
**Family has household water connection [N = 401]**		64.5%

* Age at study child's birth.

There were 3973 diarrheal episodes observed during the surveillence period. In 3667 (92.3%) of these, a stool sample was collected and tested in association with the episode. *Campylobacter* was isolated in 306 (8.3%). There was a low rate of co-infections between *Campylobacter species* (e.g. *C. jejuni*+*C. coli*) (Table 2), a finding in agreement with prior reports [7].

**Table 2:** Diarrhea episodes and asymptomatic stools, by *Campylobacter* species.

	Diarrheal Episodes*	Asymptomatic Stools	p-value (difference in proportions)
*Campylobacter*, total	307 (8.3%)	159 (4.9%)	p<0.001
*jejuni* only	157 (4.2%)	80 (2.4%)	p<0.001
*coli* only	93 (2.5%)	54 (1.7%)	p = 0.023
*lari* only	23 (0.6%)	12 (0.4%)	p = 0.248
*jejuni& coli*	1 (<0.1%)	-	
*coli &lari*	2 (<0.1%)	-	
*sp.*	31 (0.8%)	13 (0.4%)	p = 0.036
Not *Campylobacter*-positive	3667	3112	
Cases for which no *Campylobacter* bacteriology was performed	262	-	
Total N	3973	3271	

* Diarrheal episodes were considered associated with *Campylobacter* when at least one stool sample from the episode was culture-positive for Campylobacter. A stool sample was considered associated with the episode when it was collected during, or up to one day after, the episode. Only stool samples that could be associated with anthropometry are reported here.

Asymptomatic stools were collected quarterly.

Nearly nineteen percent (18.9%) of campylobacteriosis episodes were treated with appropriate antibiotic therapy. Treated episodes had a greater duration than untreated infections (8.1 versus 2.8 days, p<0.01), and were more frequently associated with mucus (57.7% versus 33.7% of episodes, p<0.01); however, no differences were observed in the presence of gross blood (13.6% versus 7.9% p = 0.18), reported fever (42.4% versus 33.3%, p = 0.19), or the presence of fecal leucocytes or occult blood.

No significant difference were detected in fecal leucocytes, occult blood, or technician-observed mucus between asymptomatic stools with or without *Campylobacter* (6.9% v. 6.2%, 9.2% v. 8.2%, 6.3% v 4.0%, respectively). However gross blood was higher in *Campylobacter*-positive asymptomatic stools (1.7 versus 0.5%, p = 0.03).

In contrast, fecal leucocytes, occult blood, and gross blood were more common in *Campylobacter*-positive, as compared to *Campylobacter*-negative diarrheal samples (12.9 versus 8.0%, p<0.01, 17.0% versus 11.1% p<0.01, and 9.0% versus 4.4, p<0.01, respectively). However, no differences were detected for technician-observed mucus (38.3% versus 34.8%, p = 0.214).

The incidence of *Campylobacter-*associated diarrhea peaked at 0.7 episodes/child-year, in 18 month old children (Figure 1). There was then a steep decline in the incidence of *Campylobacter* diarrhea, the rate of isolation of *Campylobacter* as a proportion of diarrheal and asymptomatic declined more gradually (Figure 1).

### Risk Factors for *Campylobacter* Infection

Nearly fifty percent (49.7%) of children had a *Campylobacter* infection during the study period. Ninety-two (21.2%) experienced campylobacteriosis but no asymptomatic *Campylobacter*; 47 experienced asymptomatic *Campylobacter* only, and 79 experienced symptomatic and asymptomatic infections. In addition to the other risk factors described below, children who experienced *Campylobacter* infection during the course of the study had a higher incidence of diarrhea overall (10.9 versus 7.6 episodes/yr among children who ever versus never had *Campylobacter*); entered the study at a younger ages (mean 19.5 versus 31.8 months), and remained in the study longer (mean 34.7 versus 22.8 months in study).

Both asymptomatic and symptomatic *Campylobacter* infections were associated with younger age, a recent history of diarrheal illness and *Campylobacter* diarrhea, and less maternal education (Table 3). Per-capita income and the presence of a household water connection were predictive of *Campylobacter* diarrhea, but not asymptomatic infection (Table 3). Child sex, maternal age, breastfeeding status, seasonal effects, the presence or a private family latrine, birthweight, and the presence of poultry in the household, were not found to predict either symptomatic *Campylobacter* incidence or asymptomatic *Campylobacter*, and per-capita income and the presence of a household water connection were found to predict *Campylobacter* diarrhea, but not asymptomatic *Campylobacter* infection (Table 2).

**Table 3:** Poisson model of risk factors for *Campylobacter.*

		Incidence Rate Ratio (IRR) (univariate) Asymptomatic Campylobacter	Incidence Rate Ratio (IRR) (univariate) Campylobacter diarrhea	Incidence Rate Ratio (IRR) (multivariate) Campylobacter diarrhea
**Age**		1.07***	1.04*	1.01
		(1.02, 1.13)	(1.00, 1.08)	(0.96, 1.05)
**Age >18 months**		0.94***	0.97***	0.98
		(0.91, 0.98)	(0.94, 0.99)	(0.96, 1.01)
**Age >36 months**		0.987	0.979***	0.989
		(0.972, 1.003)	(0.969,0.990)	(0.977, 1.002)
**Stunted (ref = HAZ>−2)**		1.05	1.27*	1.18
		(0.76, 1.45)	(0.96, 1.68)	(0.90, 1.54)
**WHZ**	**> = 0**	ref	ref	ref
	**0 to −1**	0.84	1.19	0.96
		(0.53, 1.32)	(0.86, 1.65)	(0.68, 1.36)
	**<−1**	1.55	1.31	0.83
		(0.63, 3.78)	(0.62, 2.77)	(0.38, 1.82)
**non-** ***Campylobacter*** ** diarrhea**	**0 episodes**	ref	ref	ref
**in past 3 months**	**1–2 episodes**	1.41*	2.77***	2.01***
		(0.98, 2.03)	(2.02, 3.80)	(0.68, 1.36)
	**3+ episodes**	2.32***	3.94***	2.44***
		(1.42, 3.77)	(2.67, 5.83)	(1.61, 3.71)
***Campylobacter*** **-diarrhea**	**not present**	ref	ref	ref
**in past 3 months**	**present**	3.29	1.94***	1.94***
		(2.16, 5.01)	(1.33, 2.82)	(1.43,2.83)
**Natural log per-capita income**		0.90	0.72***	0.80**
		(0.73, 1.11)	(0.59, 0.88)	(0.67, 0.97)
**Some secondary maternal education**		0.63***	0.72**	0.79
**(ref = primary education or less)**		(0.45, 0.88)	(0.52, 1.00)	(0.59, 1.05)
**indoor household water connection**		0.90	0.55***	0.68***
**(ref = water source outside house)**		(0.64, 1.25)	(0.40, 0.75)	(0.51, 0.90)

* significant at <0.100 level,

** significant and <0.050 level,

*** significant at <0.010 level.

Sex, seasonal terms, maternal age, breastfeeding status, birth weight, private household latrine, and the presence of household poultry, were not significant in any model and are not shown.

The association between prior nutritional status and prior diarrhea, and prior *Campylobacter*, were examined via correlations and kappa statistics and found to be low (rho<0.05).

Stunting was a significant risk factor for *Campylobacter* diarrhea when recent diarrhea was not also included in the model. When recent diarrhea was included, the association of stunting with campylobacter risk decreased from an IRR of 1.270 compared to non-stunted children, to an IRR of 1.175. Stunting and recent diarrhea were not strongly correlated (rho = 0.050).

### Effects of *Campylobacter* Infection on Growth

Asymptomatic *Campylobacter* and *Campylobacter-*associated diarrhea were each associated with reduced weight gain over a three-month period (65.5 and 43.9 grams less weight gain, respectively) (Table 4).

**Table 4:** Association between *Campylobacter* and weight gain.

		Weight Gain (grams/3months)	
		Model 1	Model 2
***Campylobacter*** ** category**	**No ** ***Campylobacter*** ** detected**	ref	ref
	**Asymptomatic ** ***Campylobacter***	−65.5**	−67.7**
		(−128.0, −3.0)	(−130.1, −5.2)
	**Symptomatic ** ***Campylobacter,*** ** overall**	−43.9**	N/A
		(−87.6, −0.1)	
	**Symptomatic ** ***Campylobacter,*** ** untreated**	N/A	−11.0
			(−60.4, 38.5)
	**Symptomatic ** ***Campylobacter,*** ** treated**	N/A	−167.2***
			(−267.4, −67.0)
	**Non-** ***Campylobacter*** ** diarrhea**	−22.7***	−22.9***
		(−35.3, −10.1)	(−35.4, −10.4)

*significant at the p< = 0.10 level.

** significant at the p< = 0.05 level.

*** significant at the p< = 0.01 level.

Weight models adjusted for stunting at onset of interval, WHZ category at onset of interval, season, age, birth weight and per capita income.

Fractional polynomial age term 1: 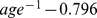; age term 2: 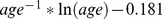 where *age* is the child's age, in days, divided by 1000.

In model 1, *Campylobacter*-related variables were asymptomatic *Campylobacter*, and symptomatic (diarrhea-associated) *Campylobacter* (2 variables).

In model 2, *Campylobacter-*related variables were asymptomatic *campylobacter*, symptomatic treated *campylobacter*, and symptomatic untreated *campylobacter* (three variables).

Symptomatic *Campylobacter,* but not asymptomatic *Campylobacter* infection, was marginally associated with reduced linear growth over nine-month intervals (Table 5). Each symptomatic *Campylobacter* episode was marginally associated with 0.059 cm less linear growth, but this was not significant at the p<0.05 level (p = 0.054).

**Table 5:** Association between *Campylobacter* and linear growth.

		Height (cm/9months)	
		Model 1	Model 2
***Campylobacter*** ** category**	**No ** ***Campylobacter*** ** detected**	ref	ref
	**Asymptomatic ** ***Campylobacter***	−0.01	−0.01
		(−0.09, 0.07)	(−0.09, 0.07)
	**Symptomatic ** ***Campylobacter,*** ** overall**	−0.06*	N/A
		(−0.12, 0.01)	
	**Symptomatic ** ***Campylobacter,*** ** untreated**	N/A	−0.03
			(−0.10, 0.03)
	**Symptomatic ** ***Campylobacter,*** ** treated**	N/A	−0.17**
			(−0.31, −0.03)
	**Non-** ***Campylobacter*** ** diarrhea**	−0.04***	−0.04***
		(−0.06, −0.02)	(−0.06, −0.03)

* significant at the p = 0.10 level.

** significant at the p = 0.05 level.

*** significant at the p = 0.01 level.

Height models adjusted for stunting at onset of interval, WHZ category at onset of interval, season, age, birth weight and per capita income.

Fractional polynomial age term 1: 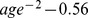; age term 2: 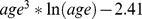 where *age* is the child's age, in days, divided by 1000.

In model 1, *Campylobacter*-related variables were asymptomatic *Campylobacter*, and symptomatic (diarrhea-associated) *Campylobacter* (2 variables).

In model 2, *Campylobacter-*related variables were asymptomatic *campylobacter*, symptomatic treated *campylobacter*, and symptomatic untreated *campylobacter* (three variables).

When subdivided into treated episodes (in which the child was still symptomatic at the time of a bacteriological confirmation of *Campylobacter*) and untreated episodes (in which the episode resolved before bacteriological confirmation), most of the impact of *Campylobacter* on weight gain was clustered among the more severe treated episodes (167.2 grams versus 11.0 grams deficit, p-value for difference <0.01). Similarly, the effect of treated *Campylobacter* on linear growth was five times greater than that of untreated symptomatic *Campylobacter* (−0.169 versus −0.034 cm deficit, p-value for difference = 0.091).

## Discussion

Both symptomatic and asymptomatic *Campylobacter* were associated with reduced weight gain over three-month periods. Additionally, symptomatic *Campylobacter* was marginally associated with reduced linear growth over nine-month periods. More severe *Campylobacter* episodes were associated with greater deficits in both weight gain and linear growth.

Because severe episodes were more likely to be treated, treatment was used to define severity in this analysis. However, it is also a confounding factor in estimating the true effect of severe campylobacteriosis on growth. Treatments were effective; 80.8% of isolates were susceptible to erythromycin, and 91.9% to azithromycin. Episodes ended on average 1.45 days after initiation of antibiotic therapy, which is 1.32 days earlier than episodes of comparable prior duration which remained untreated (data not shown). This is similar to other published reports [21].

Fifty-one percent of *Campylobacter-*associated diarrheal episodes were related to *C. jejuni,* and 30.4 percent to *C. coli.* Our decision to pool these species during the analysis was based on the limited number of samples available from each, and preliminary explorations of the data did not suggest any differences in association between *C.jejuni* and *C.coli* infections and growth. We cannot draw conclusions about differential effects of *Campylobacter* by species.

The association between co-infections and growth faltering is an interesting area of current research. We considered whether helminth infections might also have influenced growth. As previously reported, children were dewormed very frequently in this community, both due to own study protocols and parental initiative. Perhaps as a result, we found little association between helminth infections and growth [15]. The number of instances where *Campylobacter* and a helminth were identified in the same stool was low, and we were unable to relate recent helminth infection (e.g. detected in the 0–3 months prior) and *Campylobacter* infections for the same reason, almost all detected helminths were treated very promptly. As a result, this particular co-infection is unlikely to have confounded the Campylobacter-growth relationship, and we are also unable to determine whether such co-infections have a greater association with growth faltering than either infection alone.

Our analysis looked at the impact of *Campylobacter* infection on growth velocity (that is, the change in growth over 3 and 9 month periods) rather than a child's attained weight or height, or absolute risk of stunting. This approached was necessitated by the data available; the cohort was not a birth cohort, and anthropometry and surveillance histories were not available from birth for most children. However, because growth is naturally a non-linear process, natural variability from period to period may attenuate the effects of diarrheal illness over short periods [22]. Having said that, the modeling the effects of infection on short-term growth velocities remains worthwhile, especially in cohorts where anthropometric indices clearly indicate progressively growth faltering.

While there is good evidence that diarrhea as a non-specific illness, leads to reduced child growth and poorer nutritional status [23], there is a paucity of evidence associating specific enteropathogens with reduced growth. Protozoal infections, including *Cryptosporidium* and *E. histolytica*, have been found to have large negative impacts [24]–[26], with a first cryptosporidium infection associated with 0.95 cm shorter mean height one year after the infection [25]. Among enteric bacterial pathogens, ETEC-diarrhea has been shown to negatively impact weight gain, and *Shigella* to negatively impact linear growth, with an effect size of 0.073 cm per percent of days of *Shigella*-associated diarrhea, over a year [27]. Our finding that an episode of *Campylobacter,* of average duration 3.8 days, was marginally associated with nine-month deficits in height in the range of 0.059 cm, suggest effects similar in magnitude to other bacterial infections. In this cohort, *Campylobacter* was isolated in diarrheal stools as frequently as *Shigella*, and slightly less frequently than Enterotoxigenic *E. coli* (approximately 8.3%, 8.2%, and 10.1% of diarrheal episodes respectively).

When examining our data in a non-longitudinal (i.e. cross sectional) manner, we observed more frequent *Campylobacter* infections among children with lower HAZ and WHZ, in agreement with prior reports [7], [8]. However, in longitudinal models, prior WHZ failed to be predictive of campylobacteriosis, and prior stunting was only predictive of campylobacteriosis when recent diarrheal disease was not adjusted for in the analysis. In this cohort, stunting was common, while wasting, underweight, or severe undernutrition in any form, were rare. As the risk of diarrheal disease may be greatest among severely malnourished children, our population may be less than ideally suited to disentangling this effect. Similarly, younger infants (less than two years) were relatively underrepresented in this cohort, which limited our ability to test whether the association between *Campylobacter* and growth was mediated by age.

Recently, Gupta and colleagues reported that samples from a malnourished child found enteric pathogens in the family Campylobacteraceae 35-fold higher than in those of a well-nourished child [28]. This finding supports theories that *Campylobacter* carriage may be related to some underlying pathology, such as perturbed gut microbial populations, preexisting intestinal injury, or undernutrition-related immunosuppression [7]. Our finding that asymptomatic *Campylobacter* infection was associated with reduced weight gain suggests it may be a signal that a child is at risk of entering into a negative cycle of an accelerating incidence of diarrhea with the predicable negative impacts on linear growth.

It is often advised that diarrhea caused by *Campylobacter* does not warrant treatment, unless the patient is pregnant or HIV+ or the episode is associated with dysentery, fever or other severe symptoms [10]. Our finding that *Campylobacter* is associated with deficits in growth is an argument for broader treatment among children under the age of five, as well as a justification for the development of field-ready rapid diagnostics for early *Campylobacter jejuni* and *coli*.

The magnitude of the linear growth deficit attributable to Campylobacter infection is modest. However, acquired growth deficits in early childhood are associated with a variety of long-term negative outcomes including poorer cognitive development [29], lower adult work capacity and income [30], and poorer pregnancy outcomes [31], making growth deficits an accepted surrogate of human potential lost. Our findings reinforce the importance of controlling *Campylobacter* infection. Prioritization of interventions and strategies against campylobacteriosis, such as the development of rapid diagnostics for early detection at the point of care, and the development of vaccines for prevalent *Campylobacter* serotypes, may improve child growth in the developing world.
